# Associations between post-operative rehabilitation of hip fracture and outcomes: national database analysis

**DOI:** 10.1186/s12891-018-2093-8

**Published:** 2018-07-09

**Authors:** Bowen Su, Roger Newson, Harry Soljak, Michael Soljak

**Affiliations:** 1 Department of Primary Care & Public Health, School of Public Health, Imperial College London, W6 8RP, London, UK; 2 Department of Anaesthetics, St Peter’s Hospital, Chertsey, KT16 0PZ UK; 3 Centre for Population Health Sciences (CePHaS), Lee Kong Chian School of Medicine, Nanyang Technological University, Singapore, 308232 Singapore

**Keywords:** Hip fracture, Rehabilitation, Physical therapy, Clinical audit

## Abstract

**Background:**

Rehabilitation programmes are used to improve hip fracture outcomes. There is little published trial clinical trial or population-based data on the effects of the type or provider of rehabilitation treatments on hip fracture outcomes. We evaluated the associations of rehabilitation interventions with post-operative hip fracture outcomes.

**Methods:**

Cross-sectional (2013–2015) analysis of data from the English National Hip Fracture Database (NHFD) from all 191 English hospitals treating hip fractures. Of 62,844 NHFD patients, we included 17,708 patients with rehabilitation treatment and 30-day mobility data, and 34,142 patients with rehabilitation treatment and discharge destination data. The intervention was early mobilisation rehabilitation treatments delivered by a physiotherapist (PT, physical therapist in North America) or other clinical staff as identifiable in NHFD. We used ordinal logistic and propensity scoring regression models to adjust for confounding variables including age, sex, pre-fracture mobility, operative delay, and cognitive function and peri-operative risk scores.

**Results:**

In both the adjusted multivariate and propensity-weighted analyses, mobilisation on the day or the day following surgery is associated with better mobility function 30 days after discharge. However patients mobilised by a PT did not have better mobility compared to mobilisation by other professionals. Patients who received a PT assessment were not protected from poorer mobility 30 days after discharge, compared with those who did not receive an assessment. The discharge destination outcome is also better in mobilised than unmobilised patients, whether done by a PT or another health professional, and the difference persists, slightly attenuated, after propensity weighting.

**Conclusions:**

In addition to the type of health professional initiating mobilisation, data on rehabilitation treatment activity and post-operative gait speed is needed to determine optimum rehabilitation dosage and functional outcome. After adjustment patients mobilised by non-PTs did as well as patients mobilised by PTs, suggesting that PTs’ current roles in very early rehabilitation should be reconsidered, with a view to redeploying them to more specialised later rehabilitation activity.

**Electronic supplementary material:**

The online version of this article (10.1186/s12891-018-2093-8) contains supplementary material, which is available to authorized users.

## Background

Hip fractures in later life result in a high morbidity and mortality rate, with an often permanent decline in mobility, independence and quality of life [1]. Over a third of patients will have died 1 year after the fracture, compared with an expected annual mortality of about 10% in this age group [2]. One-year mortality after hip fracture has declined over the last decade in the United Kingdom (UK), [3] but the three-fold difference in one-year mortality between hip fracture patients and the general population has remained.

Although surgery is generally successful, few people recover fully, and there is a significant impact on their quality of life. Most survivors fail to regain former levels of mobility and activity, many become more dependent, and around 10% are unable to return to their previous residence [1, 4]. Many older people often already have loss of skeletal strength from osteoporosis, and sarcopenia, which is in itself a risk factor for falls and fractures [5]. In addition, people suffering a hip fracture frequently have other medical and physical problems, including impaired physical and cognitive function [6, 7]. There is also a significant psychological effect of hip fracture [8].

A variety of post-operative rehabilitation programmes are used to improve mobility, maximise physical function and prevent or reverse physical deconditioning. A 2009 Cochrane review of inpatient multi-disciplinary rehabilitation found a lack of evidence regarding what components of rehabilitation were essential for mobility recovery [9]. A 2010 Cochrane review of rehabilitation interventions for improving physical and psychosocial functioning included nine small heterogeneous trials involving 1400 participants [10]. It found conflicting results for specialist-nurse led care and educational and motivational interventions. A 2011 Cochrane review of interventions for improving mobility after hip fracture included 19 trials involving 1589 older adults, but these were mainly small, and often with methodological flaws [1]. Several recent studies in small populations evaluated the effectiveness of physiotherapy interventions in people with hip fracture [11–14]. However there has been little published rehabilitation research using large population-based datasets, [15] and rehabilitation data in the English National Hip Fracture Database Audit (NHFD) has not been extensively analysed previously. Furthermore, the results of randomised controlled trials are not necessarily replicated in “real world evidence” from unselected populations. The primary objectives of our evaluation were to use recent data from the [3] to:

- evaluate, after appropriate case-mix adjustment, the associations of rehabilitation (including physiotherapy) interventions with post-operative hip fracture mobility
- evaluate the associations of rehabilitation interventions with postoperative discharge destination and return to the same setting as pre-fracture.

The secondary objectives were:

- To assess the data quality of relevant NHFD variables
- To assess the appropriateness of current NHFD variables to audit rehabilitation interventions and to make recommendations for improvement

## Methods

### Data source

The NHFD is a clinically-led, web-based audit of hip fracture care and secondary prevention in England, Wales, Northern Ireland and the Channel Islands. All 253 eligible hospitals who treat hip fractures are registered with NHFD, and 98% participate by regularly uploading case records in a standard dataset format. All patients aged 60 and over with a hip fracture coded as S72.0, S72.1, S72.2 in ICD-10, including subtrochanteric fractures, are submitted, regardless of the fracture mechanism. The NHFD is approved by the NHS England Health Research Authority Confidentiality Advisory Group to collect patient data without consent under Section 251 exemption, so no ethical approval was required for this study. We carried out the same data quality checks used by NHFD on the 2013–15 data we received [16].

### Exposure variables

To measure the effects of rehabilitation it would be most useful to have an activity type variable which described the type of rehabilitation delivered by specialist, mainly PT staff and the intensity e.g. number of sessions. As this is not part of the NHFD dataset we were forced to use proxies for rehabilitation activity or exposure. We used “mobilised on day of or day following surgery” as one measure of rehabilitation exposure. It is recognised that not all patients will be suitable for such mobilisation, and this field only seeks to capture how quickly individuals progress in initial physiotherapy. A patient would be described as ‘mobilised’ if they are able to sit or stand out of bed on the day of their return from operation, or on the following day. We used the variable “assessed by PT on day of or day after surgery” as another measure of rehabilitation exposure. Unfortunately these two variables are optional so some data is missing.

### Predictor variables

We used demographic variables known to be associated with hip fracture outcomes, including age, sex, and delay from admission to operation. The Abbreviated Mental Test Score (AMTS), which is assessed pre-operatively, is a simple and robust 10 item screening tool for acute patients. In AMTS poor cognitive function = 0 and good cognitive function = 10. The American Society of Anaesthesiologists (ASA) score is a preoperative risk score based on the presence of co-morbidities at the time of surgery. An ASA score > 2 is associated with increased risk additional to that of classification of operation and duration of surgery.

### Outcome variables

The first outcome variable was 30-day mobility score on a scale from 1 to 5, which counter-intuitively is higher for less mobile patients in NHFD. A new single mobility score has recently (2015) replaced all four measures previously used by the NHFD. In order to replace missing values of the single mobility score in older data, we used the translational matrix shown in Additional file 1: Table S1 in the online Supplementary Data file.

The second outcome variable was discharge destination from acute orthopaedic ward in combination with the location from which the patient was admitted. For the preoperative setting we used “admitted from”. The method for combining the two variables is shown in Additional file 1: Table S2. Discharge destination models were fitted using the 30-day discharge destination combined with preoperative setting expressed on an integer scale from 1 to 4, with higher integers indicating better outcomes (1 = “Dead”, 2 = “Worse”, 3 = “Same”, 4 = “Better”).

### Statistical analysis

Ordinal logistic regression models can be used where the outcome variable is nominal or ordinal i.e. with more than two levels [17, 18]. In mathematical notation:

$$ L\left(\upbeta \right)=\_n\sum i=1\;y1 ig1\left(\mathrm{x}i\right)+y2 ig2\left(\mathrm{x}i\right)\hbox{-} \mathrm{In}\left(1+\mathrm{e}g1\left(\mathrm{x}i\right)+\mathrm{e}g2\left(\mathrm{x}i\right)\right). $$

We tested all the variables of interest in the NHFD dataset to check whether they were statistically associated with discharge destination (χ^2^ tests and likelihood ratio tests). These variables were used to fit the ordinal logistic regression models. We used the *predict* command in Stata 14 to compute the ordinal-logistic odds ratio. We computed the c-statistic for a predictive score to measure the performance of the model.

Propensity score methods can be used for confounding control in non-experimental research [19]. We also carried out a propensity scoring or matching analysis, which is often used to obtain a better estimate of effect size in real-world examples. Propensity score models do not rely on modelling the outcome, but on a model of the treatment given the confounders [20, 21].

The mobility outcome was mobility at 30 days post-operatively. The Mobility models were fitted by using complete cases only. The treatment or exposure variable was mobilisation on the day of or day after surgery. Confounders were gender, age at discharge, hours elapsed between admission and surgery, pre-operation AMTS, ASA grade, and pre-fracture mobility score (1 to 5 or missing).

We defined a propensity score for mobilisation treatment with respect to confounders, using a logistic regression model, then defined weights to estimate the average treatment effect (ATE), [21] equal to the reciprocal of the fitted probability of mobilisation for the mobilised and non-mobilised patients respectively. More information on the propensity scores, weights and balance checks is provided in the online Additional file 1: Figures S3-S5. Having defined the propensity weights, we used Poisson regression models, with Huber variances, to regress the 30-day mobility score with respect to the binary treatment variable (mobilisation), with two mean mobility scores for mobilised and unmobilised patients, respectively. The first model was unadjusted/unweighted, and the second model was weighted using the ATE weights. For both models, we estimated the ATE using the Stata 14 add-on package *scenttest*, [22] which carries out a scenario *t*-test between the mobilised and unmobilised scenarios. We carried out balance checks for the propensity score using the Stata add-on package *somersd* [23].

Discharge destination models were fitted as for Mobility. We defined a propensity score for mobilisation treatment with respect to confounders, using a multinomial logistic regression model. For each patient, we defined three propensity scores, one per mobilisation group, as the fitted probability of the patient being in the mobilisation group. We then defined weights for estimation of the ATE [21]. Therefore we were directly standardising the sample to a hypothetical target population, with the same distribution of covariates as the sample, but with no association between treatment-propensity and treatment. The unadjusted Somers’ *D* suggested that our ATE weights had balanced the propensity score. We then used Poisson regression models and the Stata packages as for the mobility outcome.

## Results

Patient numbers are shown in the flowchart in Additional file 1: Figure S1. Of a total of 62,844 patients, there were 17,708 patients with non-missing values both for the treatment exposure and for 30-day mobility, and 34,142 patients with non-missing values both for the treatment variable and for the discharge-destination outcome, because as the latter did not require contacting patients but could be obtained from electronic health records (EHRs) it was easier to collect. Therefore, all analyses were subject to the caution of high missingness levels for the treatment exposure and the outcome.

### Mobility

Most of the baseline characteristics of the complete cases in the Mobility model population after data translation are shown in Table 1. For further background, Additional file 1: Table S3 in the online Supplementary Data shows the mobility characteristics of the whole (both complete and incomplete cases) Mobility model outcome population before data translation. For comparison, Additional file 1: Table S4 shows the baseline characteristics of mobility variables before and after mobility data translation. Additional file 1: Table S5 shows all the baseline characteristics of Mobility Model population after data translation, for complete cases only.

**Table 1:** Key baseline characteristics of complete cases of the Mobility model population after data translation

Variables	Mobility model	
	Frequency	Percentage %
Age group		
60–69	1681	7.3
70–79	4384	19.0
80–89	10,471	45.5
> 90	6504	28.2
Total	23,040	100.0
Sex		
Male	6228	27.0
Female	16,812	73.0
Total	23,040	100.0
Pre Fracture Mobility		
Freely mobile without aids	7939	34.5
Mobile outdoors with one aid	4646	20.2
Mobile outdoors with two aids or frame	2619	11.4
Some indoor mobility but never goes out	7132	31.0
No functional mobility	391	1.7
Missing	313	1.4
Total	23,040	100.0
Mobility at 30 days		
Freely mobile without aids	452	2.0
Mobile outdoors with one aid	1746	7.6
Mobile outdoors with two aids or frame	3759	16.3
Some indoor mobility but never goes out	14,032	60.9
No functional mobility	3051	13.2
Missing	0	0.0
Total	23,040	100.0
Physiotherapy assessment		
No	399	1.7
Yes	22,546	97.9
Missing	95	0.4
Total	23,040	100.0
Mobilised on day of or day following surgery		
No	3332	14.5
Yes-physiotherapy	13,210	57.3
Yes-Other	661	2.9
Missing	5837	25.3
Total	23,040	100.0

Χ^2^ tests (Additional file 1: Table S1) showed patients’ pre-fracture mobility levels were most strongly associated with mobility 30 days after discharge, and AMTS, ASA grade and patients’ age were also strongly associated with post fracture mobility. Gender and whether patients received PT assessment were weakly associated with post fracture mobility.

Table 2 shows the univariate and multivariate ordinal logistic regression analyses of the mobility model (as noted in the Methods a higher score indicates worse mobility). After adjustment, mobilisation on the day or the day following surgery is associated with a lower mobility score i.e. better mobility function 30 days after discharge. In the univariate analysis, mobilisation by a PT day or the day following surgery was more protective from a high score than mobilisation by other health professionals. However, in the adjusted/multivariate analysis, patients mobilised by a PT were not protected from poorer mobility compared to mobilisation by other professionals. Similarly, in both analyses, patients who received a PT assessment were not protected from poorer mobility 30 days after discharge, compared with those who did not receive an assessment.

**Table 2:** Univariate & multivariate ordinal logistic analysis for mobility (NB higher score = less mobile) 30 days after discharge model

	Univariate Analysis		Multivariate Analysis	
	ORs (95% CIs)	*P* value	ORs (95% CIs)	*P* value
Age 60–69	1		1	
70–79	1.067 (1.036–1.099)	< 0.001	1.139 (0.936–1.388)	0.196
80–89	0.857 (0.842–0.872)	< 0.001	1.628 (1.310–2.024)	< 0.001
> 90	1.513 (1.876–2.778)	< 0.001	2.269 (1.865–2.760)	< 0.001
Sex Male	1		1	
Female	1.018 (1.470–1.558)	0.54	0.931 (0.877–0.989)	0.02
Mobilised day of/after surgery No			1	
Yes-PT	0.438 (0.415–0.463)	< 0.001	0.541 (0.511 0.573)	< 0.001
Yes-Other	0.354 (0.304–0.413)	< 0.001	0.472 (0.403–0.553)	< 0.001
ASA grade Normal healthy individual	1		1	
Mild systemic disease that does not limit activity	2.627 (2.241–3.080)	< 0.001	1.601 (1.359–1.885)	< 0.001
Severe systemic disease that limits activity but is not incapacitating	6.987 (5.970–8.178)	< 0.001	2.453 (2.081–2.892)	< 0.001
Incapacitating systemic disease which is constantly life-threatening	12.096 (10.184–14.368)	< 0.001	3.078 (2.567–3.690)	< 0.001
Moribund - not expected to survive 24 h with or without surgery	36.562 (20.941–63.834)	< 0.001	7.057 (3.824–13.025)	< 0.001
Unknown	8.536 (6.734–10.820)	< 0.001	2.35 (1.828–3.021)	< 0.001
AMTS Score Poor cognitive function 0	1.263 (1.055–1.511)	0.084	1.179 (0.979–1.420)	0.084
1	1		1	
2	0.906 (0.723–1.136)	0.339	0.896 (0.710–1.132)	0.339
3	0.858 (0.683–1.077)	0.53	0.931 (0.736–1.178)	0.53
4	0.737 (0.589–0.923)	0.038	0.789 (0.626–0.995)	0.038
5	0.747 (0.600–0.929)	0.188	0.861 (0.687–1.079)	0.188
6	0.647 (0.522–0.802)	0.033	0.787 (0.631–0.982)	0.033
7	0.474 (0.388–0.580)	< 0.001	0.626 (0.509–0.771)	< 0.001
8	0.378 (0.313–0.456)	< 0.001	0.571 (0.470–0.693)	< 0.001
9	0.262 (0.219–0.314)	< 0.001	0.465 (0.386–0.560)	< 0.001
Good cognitive function 10	0.163 (0.137–0.193)	< 0.001	0.373 (0.312–0.446)	< 0.001
Not done	0.328 (0.266–0.405)	< 0.001	0.473 (0.382–0.587)	< 0.001
PT assessment No	1		1	
Yes	1.191 (0.996–1.424)	0.055	1.267 (1.056–1.520)	0.008
Pre Fracture Mobility Freely mobile no aids	1		1	
Mobile outdoors one aid	2.043 (1.901–2.197)	< 0.001	1.621 (1.503–1.749)	< 0.001
Mobile outdoors two aids or frame	2.813 (2.568–3.082)	< 0.001	1.956 (1.778–2.150)	< 0.001
Some indoor mobility never goes out	7.872 (7.315–8.472)	< 0.001	3.729 (3.442–4.039)	< 0.001
No functional mobility	53.419 (42.656 66.899)	< 0.001	27.849 (21.388–34.512)	< 0.001
Unknown	10.046 (7.929–12.727)	< 0.001	3.892 (3.051–4.964)	< 0.001
Delay (hours) to operation < 24	1		1	
24–48	0.304 (0.237–0.389)	< 0.001	0.999 (0.999–1.002)	0.95
48–70	0.336 (0.269–0.419)	< 0.001	0.999 (0.999–1.002)	0.95
> 70	0.338 (0.272–0.419)	< 0.001	1	

We assessed discrimination of the ordinal logistic model in the same way as a traditional logistic model, using area under the receiver operating characteristic curve [24]. We fitted three different models but there was no difference in discrimination between them (area under the receiver operating characteristic curve = 0.755).

To further investigate associations of early mobilisation we then carried out a propensity-adjusted analysis comparing 30-day mobility between mobilised and unmobilised patients i.e. a two-category outcome (by grouping the PT and non-PT mobilisation). The Somers’ *Ds* of the propensity score suggested that our ATE weights had balanced the propensity score between the treatment groups. We used the propensity weights to estimate the ATE, equal to the difference between the two scenario means, with PT and Other grouped together. The results in Additional file 1: Figure S2 show that the mobilisation-propensity score predicts mobilisation, with a Somers’ D of 0.298. The ATE-adjusted scenario means are a little closer together than the unadjusted scenario means. The outcome is better (lower mobility score) in mobilised (=Yes) patients than in unmobilised patients (=No), whether the mobilisation is done by a PT or by somebody else, and the difference persists (slightly attenuated) after propensity weighting. The unadjusted results suggest that patients mobilised by a non-PT have a lower propensity score i.e. are better mobilised than patients mobilised by a PT, but the propensity-adjusted results suggest that this crude difference may easily be due to differences in the patients. This might happen if more vulnerable patients were allocated mostly to PTs and less vulnerable patients were allocated mostly to non-PTs. More detail about propensity scores and weights is provided in the online Additional file 1: Figure S3 is a forest plot of confounder categories vs Somers’ D score which shows that ATE-weighted propensity scores for confounders are greatly reduced.

**Fig. 1:**
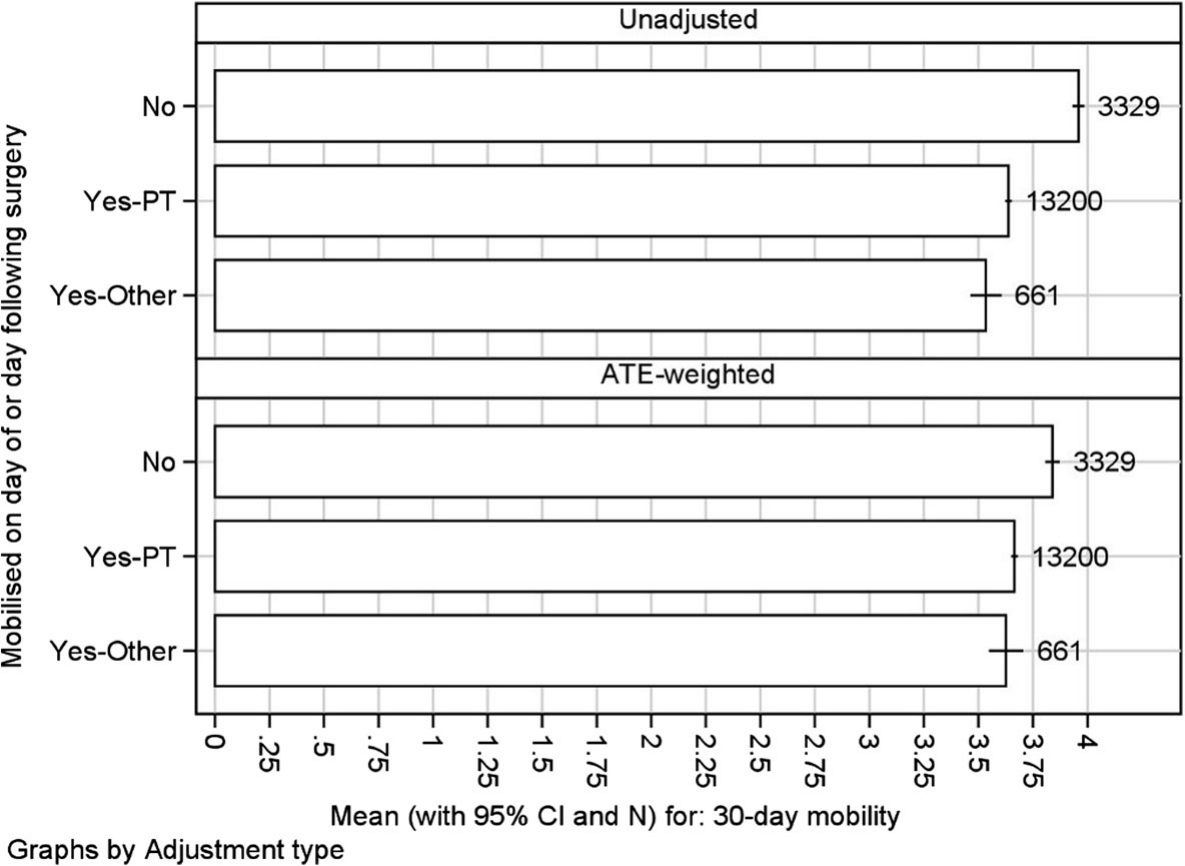
Unadjusted and adjusted difference in average treatment effect (ATE) of no mobilisation (=no) or PT mobilisation (=yes-PT) or Other mobilisation (yes-Other) on day/day after surgery for 30-day mobility outcome (Numbers of scores are shown to right of each bar)

To further investigate the associations with who performs early mobilisation we then carried out a propensity-adjusted analysis comparing 30-day mobility score between mobilised (mobilised by PT or mobilised by other professionals) and unmobilised patients i.e. using three categories to separate the effects of PTs and other staff (Fig. 1). The results in Fig. 1 show that the mobilisation-propensity score predicts mobilisation, with a Somers’ D of 0.298, as for the two-scenario analysis. The ATE-adjusted scenario means are a little closer together than the unadjusted scenario means, but not a lot. The outcome is better in mobilised patients than in unmobilised patients, whether the mobilisation is done by a PT or by somebody else. And the difference persists (slightly attenuated) after propensity weighting. However, it does not seem to make much difference whether the mobilisation is done by a PT or by somebody else, at least after propensity weighting.

### Discharge destination

**Fig. 2:**
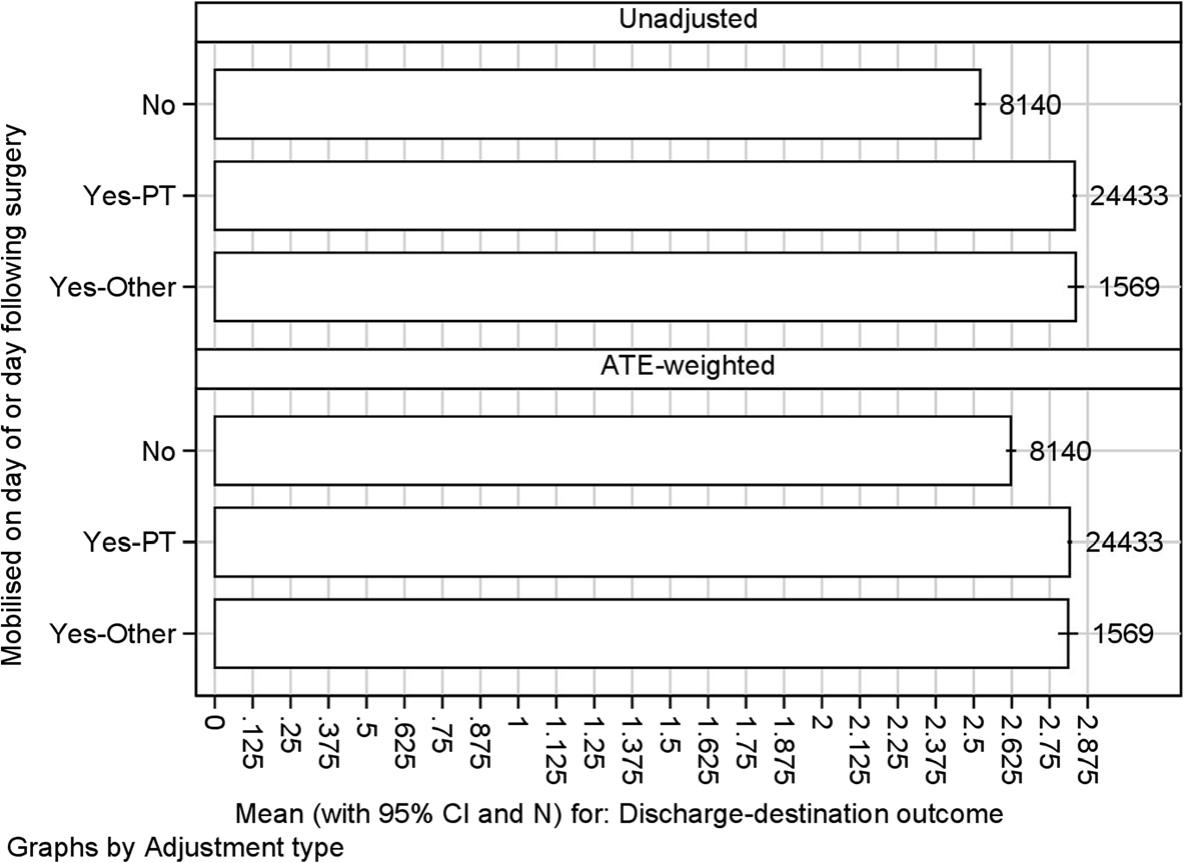
Unadjusted and adjusted difference in average treatment effect (ATE) of mobilisation on day/day after surgery for discharge-destination outcome (Numbers of scores are shown to right of each bar)

Additional file 1 Table S2 shows the baseline characteristics of the discharge destination model population. We again used propensity weights to estimate the ATEs, equal to the differences between the three scenario means (fully mobilised by PT, fully mobilised by others and fully non-mobilised) (Fig. 2). For this outcome a higher score indicates a better outcome. The mobilisation-propensity score predicts discharge destination, with a Somers’ D of 0.332. Figure 2 shows that the ATE-adjusted scenario means are a little closer together than the unadjusted scenario means. The outcome is better in mobilised than unmobilised patients, whether done by a PT or by somebody else, and the difference persists (slightly attenuated) after propensity weighting. The results are quite similar to the 30 day Mobility model, suggesting that this is a reliable association.

## Discussion

### Summary

In summary, we investigated the associations of early mobilisation, as a proxy for rehabilitation, mainly delivered by PTs, with two outcome variables, both before and after adjustment for confounding variables. In both in the 30 day mobility and discharge destination outcome models, we confirmed that patients mobilised early had a better outcome than those not mobilised early, whether they were mobilised by PTs or by non-PTs. The unadjusted analyses suggest that patients mobilised by non-PTs were less immobile at 30 days, but after adjustment, patients mobilised by non-PTs did as well as patients mobilised by PTs. There are a number of possible explanations for this: there may be no actual difference, PTs could mobilise more vulnerable patients with a worse prognosis, or non-PTs such as nurses may deliver more rehabilitation sessions. However we also showed that 57% of patients are currently being mobilised early by a PT and only 3% by “Others”, presumably nurses. If our findings are confirmed, PTs’ roles in early rehabilitation should be reconsidered, with a view to redeploying them to more effective rehabilitation activity.

There are few published cohort studies which have analysed rehabilitation exposures and outcomes, and in most instances they only measured effects of transfers to rehabilitation facilities. For example, in an Austrian cohort of 281 patients, [25] patients who were transferred to a nearby acute geriatric hospital for rehabilitation had significantly higher functional outcome scores. A study from the Scottish Hip Fracture Audit of 2708 patients aimed to determine whether place of residence is associated with a difference in access to comprehensive rehabilitation, rather than the effects of rehabilitation exposure [15]. It found that previously mobile care home patients were less likely to return to pre-admission levels of function at 120 days post fracture.

### Strengths & limitations

We used two methods and two outcomes and obtained similar results from both, suggesting that our findings are robust. However, both the models could be applied to only the sub-population of patients with complete exposure and outcome data.

Unmeasured confounding is another limitation. Although we adjusted for five confounders, it is possible that other patient factors influenced outcomes. For example, a US study was assessed the relationship between self-reported disease burden and functional improvement during and after inpatient rehabilitation [26]. Compared with patients without chronic conditions, those who had a stroke had significantly worse self-care, transfer and locomotion ratings.

The biggest limitation in our analysis is that we were forced to use binary variables for early mobilisation or PT assessment as a proxy for rehabilitation, because there is inadequate data about the process of rehabilitation in the NHFD. There is likely to be great variation in the amount of early mobilisation rehabilitation, and there is little published data on the dose-response relationship. The hip fracture guideline from the UK’s National Institute for Health & Care Excellence found only one, small randomised controlled trial with 60 patients of early versus delayed mobilisation, and three randomised studies with a total of 288 patients using three different types of PT programmes, which were not comparable and so could not be meta-analysed [27]. The American Academy of Orthopaedic Surgeons guidelines for the management of hip fractures in the elderly recommend intensive supervised occupational therapy and physiotherapy in hospital and home, [28] but the studies it quotes as evidence carried out extensive home rehabilitation programmes, [29] for example an intervention group received a median of 4.5 home visits, (PT median 3, minimum 0– maximum 7 visits; occupational therapist median 1.5, minimum 0–maximum 8 visits; and 11 patients were visited by a nurse) [30]. Real world rehabilitation may not have such a high dose.

## Conclusions

In the real world the effectiveness of interventions which were efficacious in clinical trials may be sub-optimal. Given appropriate measurement, observational research using large databases such as our study can provide valuable evidence of clinical effectiveness. We found that early mobilisation is associated with better mobility function. NHFD data collection could be improved to capture dosage of rehabilitation activity by PTs and other healthcare professionals. This should not be done by manual data entry into NHFD, but by linkage of electronic data from hospital and community-based information systems. Unfortunately there is no national dataset for paramedical activity delivered to inpatients, which would allow this data to be linked with NHFD. NHFD should ask NHS Digital to produce such a dataset. In the interim, adding two variables in NHFD to capture a meaningful numerical rehabilitation outcome such as gait speed, and rehabilitation activity such as number of sessions, should be considered. The latter would help to determine the dose-response relationship of rehabilitation.

Future research and audit should aim to confirm our finding that, after adjustment, the results of early mobilisation by nurses seems to be similar to that achieved by PTs, although this could be a result of PTs treating patients with greater morbidity or frailty.

## Additional file

Additional file 1: **Table S1.** Mobility variables translation matrix. **Table S2.** construction of admission/discharge destination outcome variable. **Figure S1.** Flowchart of mobility and discharge destination models. **Table S3** all baseline characteristics of mobility outcome population (both complete and incomplete cases) before data translation. **Table S4.** Frequency of mobility categories before and after data translation. **Table S5.** all baseline characteristics of Mobility Model population after data translation, complete cases only. **Table S6.** Strength of association between patient characteristics used in the Mobility models and mobility 30 days after discharge. **Figure S2** unadjusted and adjusted difference in average treatment effect (ATE) of no mobilisation (=no) or mobilisation (=yes) on day/day after surgery for 30-day mobility outcome. **Table S7** baseline characteristics of the discharge destination model. **Figure S3** chart of confounder categories vs Somers’ D score for two category mobility outcome. **Figure S4.** Somers’ D of propensity score and component covariates with respect to: mobilisation = No. **Figure S5.** Somers’ D of propensity score and component covariates with respect to: mobilisation==No. (DOCX 1030 kb)
