# Correction to: Associations between post-operative rehabilitation of hip fracture and outcomes: national database analysis

**DOI:** 10.1186/s12891-018-2194-4

**Published:** 2018-09-19

**Authors:** Bowen Su, Roger Newson, Harry Soljak, Michael Soljak

**Affiliations:** 1 Department of Primary Care & Public Health, School of Public Health, Imperial College London, W6 8RP, London, UK; 2 Department of Anaesthetics, St Peter’s Hospital, Chertsey, KT16 0PZ UK; 3 Centre for Population Health Sciences (CePHaS), Lee Kong Chian School of Medicine, Nanyang Technological University, Singapore, 308232 Singapore

## Correction

Following publication of the original article [1], the author reported an error in the Title. The original article has been corrected. The details of the error are as follows:

Incorrect Title:

Associations between post-operative rehabilitation of hip fracture and outcomes: national database analysis (90 characters)

Correct Title:

Associations between post-operative rehabilitation of hip fracture and outcomes: national database analysis
