# Supplementary material for: Associations between post-operative rehabilitation of hip fracture and outcomes: national database analysis
Source: BMC Musculoskelet Disord. 2018 Jul 9;19:211. doi: 10.1186/s12891-018-2093-8 (PMC6038238; doi:10.1186/s12891-018-2093-8)
Supplement: Supplementary file 1 — Table S1. Mobility variables translation matrix. Table S2. construction of admission/discharge destination outcome variable. Figure S1. Flowchart of mobility and discharge destination models. Table S3 all baseline characteristics of mobility outcome population (both complete and incomplete cases) before data translation. Table S4. Frequency of mobility categories before and after data translation. Table S5. all baseline characteristics of Mobility Model population after data translation, complete cases only. Table S6. Strength of association between patient characteristics used in the Mobility models and mobility 30 days after discharge. Figure S2 unadjusted and adjusted difference in average treatment effect (ATE) of no mobilisation (=no) or mobilisation (=yes) on day/day after surgery for 30-day mobility outcome. Table S7 baseline characteristics of the discharge destination model. Figure S3 chart of confounder categories vs Somers’ D score for two category mobility outcome. Figure S4. Somers’ D of propensity score and component covariates with respect to: mobilisation = No. Figure S5. Somers’ D of propensity score and component covariates with respect to: mobilisation==No. (DOCX 1030 kb) [file 12891_2018_2093_MOESM1_ESM.docx]

**Associations of rehabilitation with mobility and discharge destination after hip fracture: analysis of a national database**

**Additional file 1**

Table S1: Mobility variables translation matrix

| Category | 2015 definition | 2013-14 definition |
| --- | --- | --- |
| 1 | Regularly mobile outdoor without aids (or assistance) | Walking ability outdoors = “Regularly walked without aids”    -AND-  Accompanied to walk outdoors = ”No” |
| 2 | Mobile outdoors with only one aid | Walking ability outdoors = ”Regularly walked with one aid”  -AND-  Accompanied to walk outdoors = “No” |
| 3 | Mobile outdoors with 2 aids or a frame | Walking ability outdoors = “Regularly walked with two aids or frame”  -AND-  Accompanied to walk outdoors = “No” |
| 4 | Indoor mobility only, never goes out unassisted | “Regularly walked without aids”  “Regularly walked with one aid”  Walking ability “Regularly walked with two aids or more”  indoors =  -AND-  “Electric buggy”  Walking ability “Wheelchair or bedbound”  outdoors= “Never goes outdoors”  -OR-  Accompanied to walk outdoors = “Yes” |
| 5 | No functional mobility (wheelchair or assisted transfers or bedbound) | Walking ability Indoors = “Wheelchair or bedbound”  -AND-  “Electric buggy”  “Wheelchair or bedbound”  Walking ability outdoors = -OR-  “Never goes outdoors” |

Table S2: construction of admission/discharge destination outcome variable

| Admitted From | Categorisation of (long-term) setting admitted from^[[1]](#footnote-1)^ | Discharge Destination from NHS^[[2]](#footnote-2)^ | Outcome categories |
| --- | --- | --- | --- |
| Already in hospital | Nursing facility (needing healthcare pre-fracture) | Own home/sheltered housing | Better |
| Already in hospital | Nursing facility (needing healthcare pre-fracture) | Residential care | Better |
| Nursing care | Nursing facility (needing healthcare pre-fracture) | Own home/sheltered housing | Better |
| Nursing care | Nursing facility (needing healthcare pre-fracture) | Residential care | Better |
| Rehabilitation unit | Nursing facility (needing healthcare pre-fracture) | Own home/sheltered housing | Better |
| Rehabilitation unit | Nursing facility (needing healthcare pre-fracture) | Residential care | Better |
| Rehabilitation unit | Nursing facility (needing healthcare pre-fracture) | Dead | Died in hospital |
| Residential care | Home | Dead | Died in hospital |
| This hospital site | Transfer, long term setting unknown | Dead | Died in hospital |
| Acute hospital | Transfer, long term setting unknown | Dead | Died in hospital/worse |
| Already in hospital | Nursing facility (needing healthcare pre-fracture) | Dead | Died in hospital/worse |
| Nursing care | Nursing facility (needing healthcare pre-fracture) | Dead | Died in hospital/worse |
| Other | Unknown | Dead | Died in hospital/worse |
| Other hospital site of this trust | Transfer, long term setting unknown | Dead | Died in hospital/worse |
| Other hospital trust | Transfer, long term setting unknown | Dead | Died in hospital/worse |
| Own home/sheltered housing | Home | Dead | Died in hospital/worse |
| Already in hospital | Nursing facility (needing healthcare pre-fracture) | Nursing care | Same |
| Nursing care | Nursing facility (needing healthcare pre-fracture) | Acute hospital | Same |
| Nursing care | Nursing facility (needing healthcare pre-fracture) | Nursing care | Same |
| Other hospital site of this trust | Transfer, long term setting unknown | Acute hospital | Same |
| Own home/sheltered housing | Home | Own home/sheltered housing | Same |
| Own home/sheltered housing | Home | Residential care | Same |
| Rehabilitation unit | Nursing facility (needing healthcare pre-fracture) | Nursing care | Same |
| Residential care | Home | Own home/sheltered housing | Same |
| Residential care | Home | Residential care | Same |
| Own home/sheltered housing | Home | Nursing care | Worse |
| Residential care | Home | Nursing care | Worse |

Figure S1: Flowchart of mobility and discharge destination models

Total number of NHFD patients 2013-2015

(N=62,844)

Patients with 30 days after discharge mobility status data available

(N=17,708)

Patients without 30 days after discharge mobility status data available

(N=45,136)

Patients with 30 days after discharge mobility status data available after data transfer

(No. of patients used in mobility model)

(N=23,040)

Mobility data translated by using the matrix listed in Supplementary Table 1:

(N=5,332)

Patients with both location pre-admission and discharge destination data available

(N=34,142)

Mobility model

Discharge destination model

Table S3: all baseline characteristics of mobility outcome population (both complete and incomplete cases) before data translation

|  | Frequency | Percentage (%) |
| --- | --- | --- |
| Walking ability indoors pre-fracture |  |  |
| Regularly walked without aids | 7,405 | 11.8 |
| Regularly walked with two aids or frame | 3,999 | 6.4 |
| Regularly walked with one aid | 3,763 | 6.0 |
| Wheelchair or bedbound | 359 | 0.6 |
| Missing | 47,318 | 75.3 |
| Total | 62,844 | 100.0 |
| Walking ability outdoors pre-fracture |  |  |
| Regularly walked with two aids or frame | 2,294 | 3.7 |
| Regularly walked with one aid | 3,511 | 5.6 |
| Regularly walked without aids | 5,628 | 9.0 |
| Never goes outdoors | 2,041 | 3.3 |
| Wheelchair or bedbound | 1,456 | 2.3 |
| Electric buggy | 176 | 0.3 |
| Missing | 47,738 | 76.0 |
| Total | 62,844 | 100.0 |
|  |  |  |
| No | 12,982 | 20.7 |
| Yes | 1,994 | 3.2 |
| Wheelchair or bedbound | 284 | 0.5 |
| Missing | 47,265 | 75.7 |
| Total | 62,844 | 100.0 |
| Accompanied to walk outdoors pre-fracture |  |  |
| Yes | 3,578 | 6.0 |
| No | 8,284 | 13.0 |
| Never goes outdoors | 2,056 | 3.0 |
| Wheelchair or bedbound | 1,015 | 1.6 |
| Missing | 47,911 | 76.0 |
| Total | 62,844 | 100.0 |
| Walking ability indoors at 30 days post-fracture |  |  |
| Regularly walked with two aids or frame | 3,947 | 6.3 |
| Regularly walked without aids | 291 | 0.5 |
| Regularly walked with one aid | 646 | 1.0 |
| Wheelchair or bedbound | 948 | 1.5 |
| Missing | 57,012 | 90.7 |
| Total | 62,844 | 100.0 |
| Walking ability outdoors at 30 days post-fracture |  |  |
| Never goes outdoors | 3,151 | 5.0 |
| Regularly walked with two aids or frame | 1,029 | 2.0 |
| Regularly walked with one aid | 487 | 0.8 |
| Wheelchair or bedbound | 645 | 1.0 |
| Regularly walked without aids | 91 | 0.0 |
| Electric buggy | 29 | 0.0 |
| Missing | 57,412 | 91.4 |
| Total | 62,844 | 100.0 |
| Accompanied to walk indoors at 30 days post-fracture |  |  |
| No | 2,807 | 4.5 |
| Yes | 2,891 | 4.6 |
| Missing | 57,146 | 90.9 |
| Total | 62,844 | 100.0 |
|  |  |  |
| Accompanied to walk outdoors at 30 days post-fracture |  |  |
| Never goes outdoors | 3,147 | 5.0 |
| Yes | 1,318 | 2.1 |
| No | 892 | 1.4 |
| Missing | 57,487 | 91.5 |
| Total | 62,844 | 100.0 |
|  |  |  |
| Walking ability indoors at 120 days post-fracture |  |  |
| Regularly walked with two aids or frame | 1,644 | 2.6 |
| Regularly walked without aids | 777 | 1.2 |
| Regularly walked with one aid | 760 | 1.2 |
| Wheelchair or bedbound | 482 | 0.8 |
| Missing | 59,181 | 94.2 |
| Total | 62,844 | 100.0 |
|  |  |  |
|  |  |  |
| Walking ability outdoors at 120 days post-fracture |  |  |
| Wheelchair or bedbound | 640 | 1.0 |
| Regularly walked with two aids or frame | 838 | 1.3 |
| Regularly walked without aids | 359 | 0.6 |
| Never goes outdoors | 931 | 1.5 |
| Regularly walked with one aid | 828 | 1.3 |
| Electric buggy | 34 | 0.1 |
| Missing | 59,214 | 94.2 |
| Total | 62,844 | 100.0 |
|  |  |  |
| Accompanied to walk outdoors at 120 days post-fracture |  |  |
| No | 2,524 | 4.0 |
| Yes | 1,081 | 1.7 |
| Missing | 59,239 | 94.3 |
| Total | 62,844 | 100.0 |
| Accompanied To Walk 120 Indoors at 120 days post-fracture |  |  |
| Yes | 1,469 | 2.3 |
| No | 1,172 | 1.9 |
| Never goes outdoors | 939 | 1.5 |
| Missing | 59,264 | 94.3 |
| Total | 62,844 | 100.0 |

Table S4: Frequency of mobility categories before and after data translation

| Mobility variables | Before replacing | | After replacing | |
| --- | --- | --- | --- | --- |
|  | **Frequency** | Percent | **Frequency** | **Percent** |
| Pre Fracture Mobility |  |  |  |  |
| Freely mobile without aids | 16,899 | 26.9 | 21,690 | 34.5 |
| Mobile outdoors with one aid | 10,462 | 16.7 | 12,781 | 20.3 |
| Mobile outdoors with two aids or frame | 6,758 | 10.8 | 7,731 | 12.3 |
| Some indoor mobility but never goes out | 11,186 | 17.8 | 17,580 | 28.0 |
| No functional mobility | 793 | 1.3 | 1,144 | 1.8 |
| Missing | 16,746 | 26.7 | 1,918 | 3.1 |
| Total | 62,844 | 100.0 | 62,844 | 100.0 |
|  |  |  |  |  |
| Mobility 30 |  |  |  |  |
| Freely mobile without aids | 389 | 0.6 | 452 | 0.7 |
| Mobile outdoors with one aid | 1,464 | 2.3 | 1,746 | 2.8 |
| Mobile outdoors with two aids or frame | 3,339 | 5.3 | 3,759 | 6.0 |
| Some indoor mobility but never goes out | 10,353 | 16.5 | 14,032 | 22.3 |
| No functional mobility | 2,163 | 3.4 | 3,051 | 4.9 |
| Missing | 45,136 | 71.8 | 39,804 | 63.3 |
| Total | 62,844 | 100.0 | 62,844 | 100.0 |
|  |  |  |  |  |
| Mobility 120 |  |  |  |  |
| Freely mobile without aids | 1,147 | 1.8 | 1,436 | 2.3 |
| Mobile outdoors with one aid | 2,233 | 3.6 | 2,756 | 4.4 |
| Mobile outdoors with two aids or frame | 2,075 | 3.3 | 2,360 | 3.8 |
| Some indoor mobility but never goes out | 4,344 | 6.9 | 6,352 | 10.1 |
| No functional mobility | 1,058 | 1.7 | 1,529 | 2.4 |
| Missing | 51,987 | 82.7 | 48,411 | 77.0 |
| Total | 62,844 | 100.0 | 62,844 | 100.0 |

Table S5: all baseline characteristics of Mobility Model population after data translation, complete cases only

| Variables | Frequency | Percentage % |
| --- | --- | --- |
| Age group |  |  |
| 60-69 | 1,681 | 7.3 |
| 70-79 | 4,384 | 19.0 |
| 80-89 | 10,471 | 45.5 |
| >90 | 6,504 | 28.2 |
| Total | 23,040 | 100.0 |
| Walking Ability Indoors |  |  |
| Regularly walked without aids | 2,542 | 11.0 |
| Regularly walked with one aid | 1,251 | 5.4 |
| Regularly walked with two aids or frame | 1,370 | 6.0 |
| Wheelchair or bedbound | 114 | 0.5 |
| Missing | 17,763 | 77.0 |
| Total | 23,040 | 100.0 |
| Walking Ability Outdoors |  |  |
| Regularly walked without aids | 1,947 | 8.5 |
| Regularly walked with one aid | 1,160 | 5.0 |
| walked with two aids or frame | 770 | 3.3 |
| Wheelchair or bedbound | 494 | 2.1 |
| Electric buggy | 73 | 0.3 |
| Never goes outdoors | 746 | 3.2 |
| Missing | 17,850 | 77.5 |
| Total | 23,040 | 100.0 |
| Accompanied to Walk Indoors |  |  |
| No | 4,482 | 19.5 |
| Yes | 682 | 3.0 |
| Wheelchair or bedbound | 80 | 0.4 |
| Missing | 17,796 | 77.2 |
| Total | 23,040 | 100.0 |
| Accompanied to Walk Outdoors |  |  |
| No | 2,806 | 12.2 |
| Yes | 1,327 | 5.8 |
| Wheelchair or bedbound | 290 | 1.3 |
| Never goes outdoors | 753 | 3.3 |
| Missing | 17,864 | 77.5 |
| Total | 23,040 | 100.0 |
| Pre Fracture Mobility |  |  |
| Freely mobile without aids | 7,939 | 34.5 |
| Mobile outdoors with one aid | 4,646 | 20.2 |
| Mobile outdoors with two aids or frame | 2,619 | 11.4 |
| Some indoor mobility but never goes out | 7,132 | 31.0 |
| No functional mobility | 391 | 1.7 |
| Missing | 313 | 1.4 |
| Total | 23,040 | 100.0 |
| Mobility at 30 days |  |  |
| Freely mobile without aids | 452 | 2.0 |
| Mobile outdoors with one aid | 1,746 | 7.6 |
| Mobile outdoors with two aids or frame | 3,759 | 16.3 |
| Some indoor mobility but never goes out | 14,032 | 60.9 |
| No functional mobility | 3,051 | 13.2 |
| Missing | 0 | 0.0 |
| Total | 23,040 | 100.0 |
| ASA grade |  |  |
| Normal healthy individual | 535 | 2.3 |
| Mild systemic disease that does not limit activity | 6,279 | 27.3 |
| Severe systemic disease that limits activity but is not incapacitating | 12,809 | 55.6 |
| Incapacitating systemic disease which is constantly life-threatening | 2,838 | 12.3 |
| Moribund - not expected to survive 24 hours with or without surgery | 51 | 0.2 |
| Missing | 528 | 2.3 |
| Total | 23,040 | 100.0 |
| Sex |  |  |
| Male | 6,228 | 27.0 |
| Female | 16,812 | 73.0 |
| Total | 23,040 | 100.0 |
|  |  |  |
| Physical therapy assessment |  |  |
| No | 399 | 1.7 |
| Yes | 22,546 | 97.9 |
| Missing | 95 | 0.4 |
| Total | 23,040 | 100.0 |
| Abbreviated Mental Test Score Pre-operation |  |  |
| 0 | 2,789 | 12.1 |
| 1 | 607 | 2.6 |
| 2 | 661 | 2.9 |
| 3 | 633 | 2.8 |
| 4 | 699 | 3.0 |
| 5 | 767 | 3.3 |
| 6 | 839 | 3.6 |
| 7 | 1,186 | 5.2 |
| 8 | 1,900 | 8.3 |
| 9 | 3,097 | 13.4 |
| 10 | 8,867 | 38.5 |
| Not done | 956 | 4.2 |
| Missing | 39 | 0.2 |
| Total | 23,040 | 100.0 |
| Mobilised on day of or day following surgery |  |  |
| No | 3,332 | 14.5 |
| Yes-physical therapy | 13,210 | 57.3 |
| Yes-Other | 661 | 2.9 |
| Missing | 5,837 | 25.3 |
| Total | 23,040 | 100.0 |

Table S6: Strength of association between patient characteristics used in the Mobility models and mobility 30 days after discharge

| Variable | X^2^ | p-value |
| --- | --- | --- |
| ASA grade | 1800 | <0.001 |
| Pre-fracture mobility | 4700 | <0.001 |
| Physical therapist assessment | 18.25 | <0.001 |
| Mobilisation on the day or the following day of surgery | 483 | <0.001 |
| Sex | 27.45 | <0.001 |
| Age | 1600 | <0.001 |
| AMTS | 3100 | <0.001 |

Figure S2: unadjusted and adjusted difference in average treatment effect (ATE) of no mobilisation (=no) or mobilisation (=yes) on day/day after surgery for 30-day mobility outcome


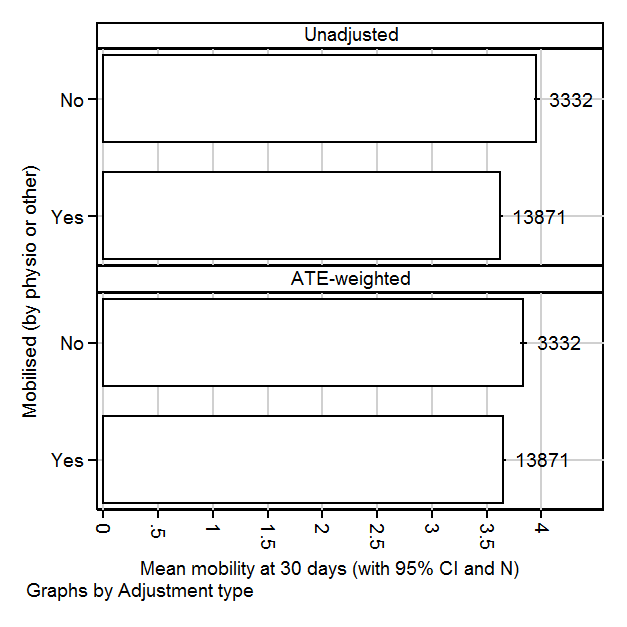


Table S7: baseline characteristics of the discharge destination model

| Variables | Frequency | Percentage % |
| --- | --- | --- |
| Age group |  |  |
| 60-69 | 3,957 | 7.5 |
| 70-79 | 10,092 | 19.1 |
| 80-89 | 23,323 | 44.2 |
| >90 | 15,412 | 29.2 |
| Total | 52,784 | 100.0 |
| Walking Ability Indoors |  |  |
| Regularly walked without aids | 6,298 | 11.9 |
| Regularly walked with one aid | 3,047 | 5.8 |
| Regularly walked with two aids or frame | 3,343 | 6.3 |
| Wheelchair or bedbound | 319 | 0.6 |
| Missing | 39,777 | 75.4 |
| Total | 52,784 | 100.0 |
| Walking Ability Outdoors |  |  |
| Regularly walked without aids | 4,810 | 9.1 |
| Regularly walked with one aid | 2,816 | 5.3 |
| walked with two aids or frame | 1,892 | 3.6 |
| Wheelchair or bedbound | 1,238 | 2.4 |
| Electric buggy | 146 | 0.3 |
| Never goes outdoors | 1,760 | 3.3 |
| Missing | 40,122 | 76.0 |
| Total | 52,784 | 100.0 |
| Accompanied to Walk Indoors |  |  |
| No | 10,788 | 20.4 |
| Yes | 1,730 | 3.3 |
| Wheelchair or bedbound | 252 | 0.5 |
| Missing | 40,014 | 75.8 |
| Total | 52,784 | 100.0 |
| Accompanied to Walk Outdoors |  |  |
| No | 6,903 | 13.1 |
| Yes | 2,997 | 5.7 |
| Wheelchair or bedbound | 843 | 1.6 |
| Never goes outdoors | 1,772 | 3.4 |
| Missing | 40,269 | 76.3 |
| Total | 52,784 | 100.0 |
| Pre Fracture Mobility |  |  |
| Freely mobile without aids | 18,457 | 35.0 |
| Mobile outdoors with one aid | 10,437 | 19.8 |
| Mobile outdoors with two aids or frame | 6,363 | 12.1 |
| Some indoor mobility but never goes out | 14,907 | 28.2 |
| No functional mobility | 1,003 | 1.9 |
| Missing | 1,617 | 3.1 |
| Total | 52,784 | 100.0 |
| Mobility at 30 days |  |  |
| Freely mobile without aids | 413 | 0.8 |
| Mobile outdoors with one aid | 1,587 | 3.0 |
| Mobile outdoors with two aids or frame | 3,534 | 6.7 |
| Some indoor mobility but never goes out | 12,346 | 23.4 |
| No functional mobility | 2,767 | 5.2 |
| Missing | 32,137 | 60.9 |
| Total | 52,784 | 100.0 |
|  |  |  |
| ASA grade |  |  |
| Normal healthy individual | 1,223 | 2.3 |
| Mild systemic disease that does not limit activity | 13,985 | 26.5 |
| Severe systemic disease that limits activity but is not incapacitating | 28,376 | 53.8 |
| Incapacitating systemic disease which is constantly life-threatening | 6,791 | 12.9 |
| Moribund - not expected to survive 24 hours with or without surgery | 210 | 0.4 |
| Missing | 2,199 | 4.2 |
| Total | 52,784 | 100.0 |
| Sex |  |  |
| Male | 14,875 | 28.2 |
| Female | 37,909 | 71.8 |
| Total | 52,784 | 100.0 |
|  |  |  |
| Physical therapy assessment |  |  |
| No | 1,845 | 3.5 |
| Yes | 50,560 | 95.8 |
| Missing | 379 | 0.7 |
| Total | 52,784 | 100.0 |
| Abbreviated Mental Test Score Pre-operation |  |  |
| 0 | 6,939 | 13.2 |
| 1 | 1,446 | 2.7 |
| 2 | 1,611 | 3.1 |
| 3 | 1,504 | 2.9 |
| 4 | 1,598 | 3.0 |
| 5 | 1,784 | 3.4 |
| 6 | 1,917 | 3.6 |
| 7 | 2,657 | 5.0 |
| 8 | 4,230 | 8.0 |
| 9 | 6,677 | 12.7 |
| 10 | 19,801 | 37.5 |
| Not done | 2,337 | 4.4 |
| Missing | 283 | 0.5 |
| Total | 52,784 | 100.0 |
| Mobilised on day of or day following surgery |  |  |
| No | 9,105 | 17.3 |
| Yes-physical therapy | 27,051 | 51.3 |
| Yes-Other | 1,736 | 3.3 |
| Missing | 14,892 | 28.2 |

**Propensity scores and weights**

The confounders included in the models as follows:

1. Gender and age. These were included using an interactive unrestricted cubic reference spline,[26] with separate spline bases for males and females, and parameters for each gender representing the value of the spline for that gender at ages 60, 65, 70, 75, 80, 85, 90, 95 and 100 years (knots were inserted interpolatively).
2. Hours elapsed between admission and surgery. These were included using an additive reference spline basis, with reference points at 0, 24 (baseline), 48, 72, 96, 120, 144 and 168 hours, plus an indicator for a missing value for hours elapsed.
3. AMTS. This was included as an additive discrete factor, with levels 0 (baseline), 1, 2, 3, 4, 5, 6, 7, 8, 9, 10, “Not done” and “Missing”.
4. ASA grade. This was included as an additive discrete factor, with values 1 (baseline), 2, 3, 4, 5, and “Missing”.
5. Pre-fracture mobility. This was included as an additive discrete factor, with values 1 (baseline), 2, 3, 4, 5 and “Missing”.

This implied a model with 18 baseline odds (corresponding to 2 values of gender combined with 9 reference ages), and 30 odds ratios (excluding the 4 omitted baseline odds ratios for hours elapsed, AMTS, ASA and pre-fracture mobility), implying 48 non-omitted parameters in all. We defined the propensity score as the fitted probability of a patient being mobilised.

We carried out balance checks for the propensity score, using the Stata add-on package *somersd* to estimate unadjusted (unweighted) and ATE-weighted versions of Somers’ *D* of the propensity score, and of its component covariates, with respect to the binary mobilisation treatment variable. We found that the unadjusted Somers’ D of the propensity score with respect to treatment was 0.298, but the ATE-weighted Somers’ D of propensity score with respect to treatment was 0.012, and the component covariates (reference splines or factor-level indicators) all had values of Somers’ D of magnitude 0.010 or less, suggesting that our ATE weights had balanced the propensity score and the component covariates between the treatment groups (see below). We used the Stata add-on package *haif*[27] to compute homoscedastic variance and standard-error inflation factors due to ATE weighting for the treatment effect in an equal-variance model, assuming that variances were equal and that the ATE weighting was not really necessary. We found that ATE weighting was not expected to inflate the variance or the standard error badly.

**Propensity score balance check for two category mobility outcome**

Supplementary Data Figure 3 is a chart of confounder categories vs Somers’ D score, which is used as a propensity balance check. This demonstrates that the propensity score should work well as an overall predictor, as ATE-weighted propensity scores for confounders are uniformly and greatly reduced.

Figure S3: chart of confounder categories vs Somers’ D score for two category mobility outcome


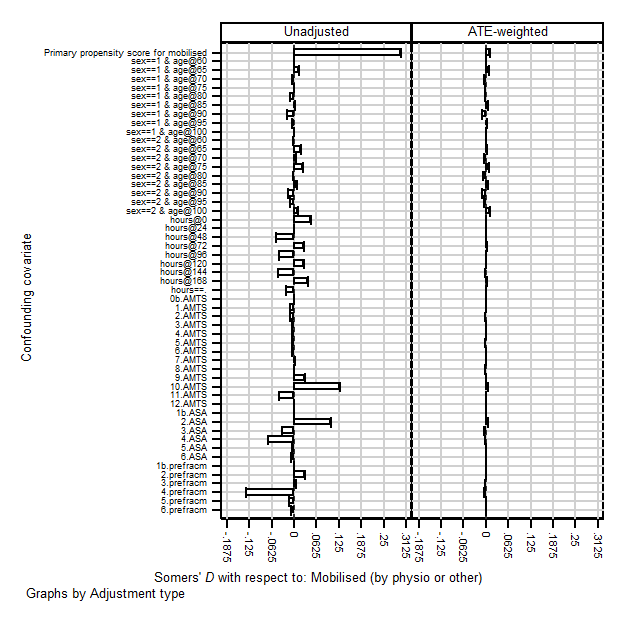


**Propensity score balance check for mobilisation as three category outcome**

To further investigate the effects of who performs early mobilisation we then carried out a propensity-adjusted analysis comparing 30-day mobility between mobilised (mobilised by PT or mobilised by other professionals) and unmobilised patients i.e. using three categories to separate the effects of PTs and other staff. Supplementary Figure 4 is a chart of confounder categories vs Somers’ D score, which is used as a propensity balance check. This demonstrates that the propensity weights should probably balance the exposure-confounder associations well.

Figure S4: Somers' D of propensity score and component covariates with respect to: mobilisation=No


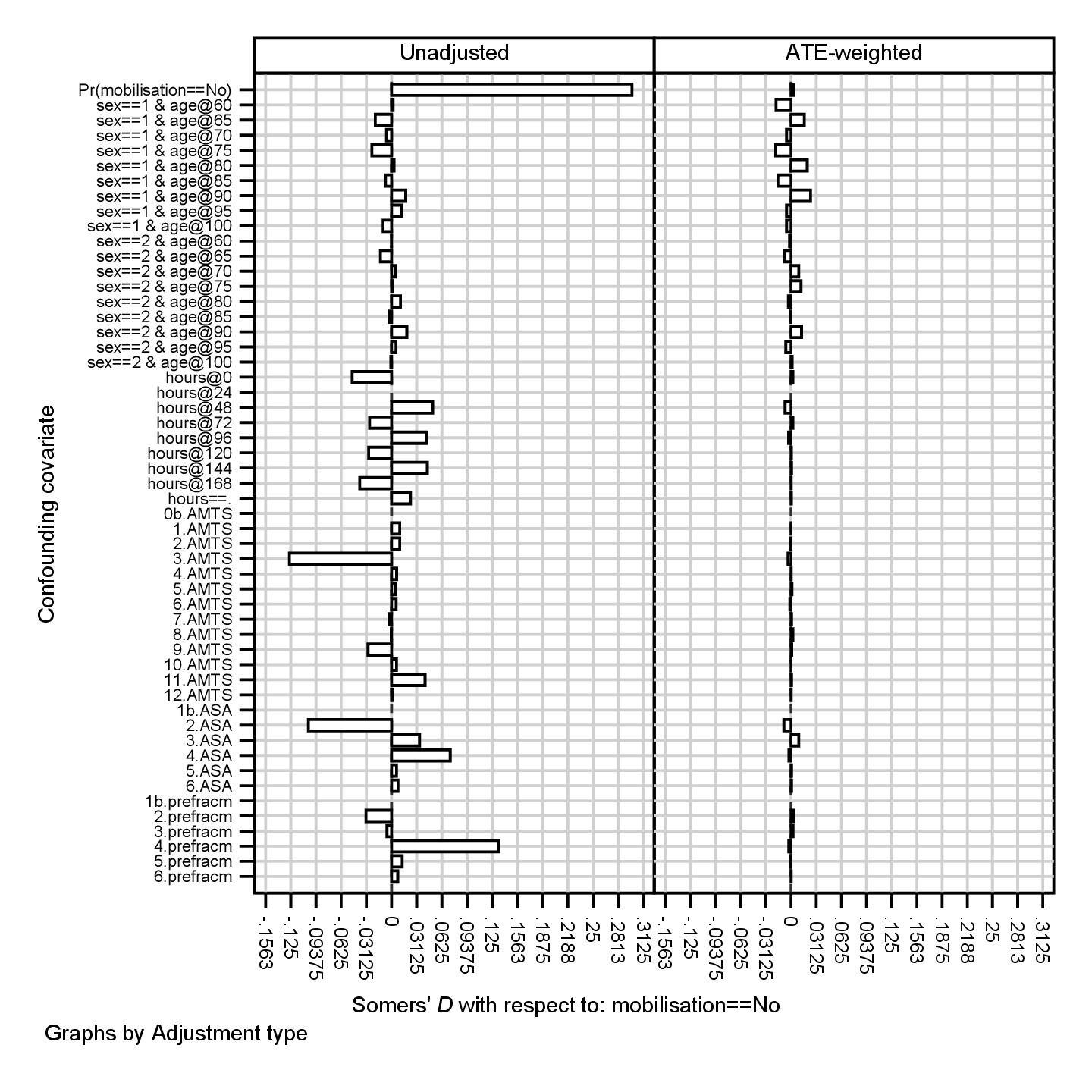


**Propensity score balance check for discharge destination outcome**

To investigate the effects of early mobilisation on discharge destination we carried out a propensity-adjusted analysis comparing discharge destination between mobilised (PT mobilised or other professionals mobilised) and unmobilised patients, using similar methods as for the mobility outcome. Supplementary Figure 5is a chart of confounder categories vs Somers’ D score, which is used as a propensity balance check. This demonstrates that the propensity score should probably work well as an overall predictor.

Figure S5: Somers' D of propensity score and component covariates with respect to: mobilisation==No


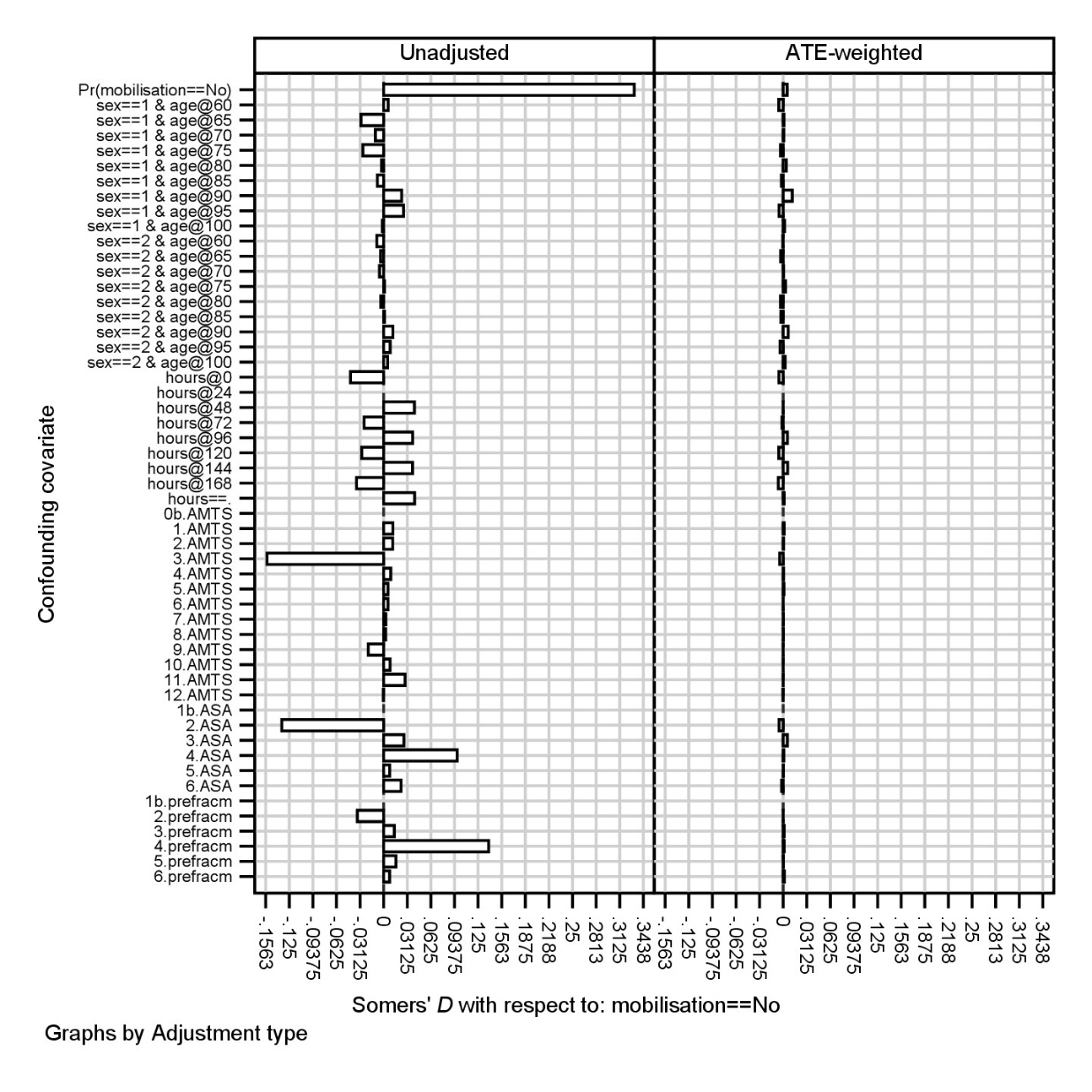


1. NHFD variable 2.07 [↑](#footnote-ref-1)
2. NHFD 6.06 [↑](#footnote-ref-2)
